# The Impact of Prolonged Storage of Red Blood Cells on Cancer Survival

**DOI:** 10.1371/journal.pone.0068820

**Published:** 2013-07-16

**Authors:** Natasha Kekre, Ranjeeta Mallick, David Allan, Alan Tinmouth, Jason Tay

**Affiliations:** 1 Department of Medicine, Division of Hematology, The Ottawa Hospital, Ottawa, Ontario, Canada; 2 Centre for Transfusion Research, The Ottawa Hospital, Ottawa, Ontario, Canada; 3 Clinical Epidemiology Program, The Ottawa Hospital, Ottawa, Ontario, Canada; 4 Regenerative Medicine Program, Ottawa Hospital Research Institute, Ottawa, Ontario, Canada; 5 Division of Hematology, Blood and Marrow Transplant Program, The Ottawa Hospital, Ottawa, Ontario, Canada; Robert Wood Johnson Medical School, United States of America

## Abstract

**Background:**

The duration of storage of transfused red blood cells (RBC) has been associated with poor clinical outcomes in some studies. We sought to establish whether prolonged storage of transfused RBC in cancer patients influences overall survival (OS) or cancer recurrence.

**Methods and Findings:**

Patients diagnosed with cancer at The Ottawa Regional Cancer Centre between January 01, 2000 and December 31, 2005 were included (n = 27,591) where 1,929 (7.0%) received RBC transfusions within one year from diagnosis. Transfused RBC units were categorized as “new” if stored for less than 14 days, “intermediate” if stored between 14 and 28 days and “old” if stored for more than 28 days. Baseline characteristics between the comparative groups were compared by ANOVA test. Categorical variables and continuous variables were compared using Chi-squared and Wilcoxan rank-sum tests respectively. Overall survival was not associated with duration of storage of transfused RBC with a median survival of 1.2, 1.7, 1.1 years for only new, intermediate and old RBC units respectively (p = 0.36). Cancer recurrence was significantly higher in patients who received a RBC transfusion than those who did not (56.3% vs 33.0% respectively; p<0.0001) but was not affected by the duration of storage of transfused RBC (p = 0.06). In multivariate analysis, lung cancer, advanced stage, chemotherapy, radiation, cancer-related surgery and cancer recurrence were associated with inferior OS (p<0.05), while age, advanced stage, lung cancer, and more than 6 units of blood transfused were associated with cancer recurrence (p<0.05). The duration of storage of RBC before transfusion was not associated with OS or cancer recurrence in multivariate analysis.

**Conclusion:**

In patients diagnosed with cancer, the duration of storage of transfused RBC had no impact on OS or cancer recurrence. This suggests that our current RBC storage policy of providing RBC of variable duration of storage for patients with malignancy is safe.

## Introduction

Red blood cell (RBC) transfusions remain an essential component in the management of medically ill patients. The goal of RBC transfusion is to increase the delivery of oxygen to tissue in vulnerable patients. [1] Currently, RBC units can be safely stored for transfusion for up to 42 days, based on studies that have optimized storage by adding nutrients, phosphate and adenine. [2]–[5] It is becoming clearer that changes that occur in RBC storage might impair oxygen delivery through a multitude of metabolic and physiologic changes that occur during storage. [6], [7] These changes in RBC storage ultimately lead to corpuscular changes in the red cell, impairing RBC deformability. Oxidative damage to the red cell membrane, depletion of 2, 3-DPG and ATP, and membrane phospholipid vessiculation contribute to corpuscular changes in the RBC during storage. [8] The sum total of this effect on the RBC is known as the “storage lesion” [8].

There has been increasing interest in exploring whether the duration of storage of RBC units independently influences clinical outcomes. [9] Studies in critically ill patients demonstrated that the age of blood transfused may adversely affect intensive care unit length of stay and overall survival (OS). [10]–[12] These studies most commonly define “new” as being stored for less than 14 days and “old” as being stored for more than 14 days. Although there is no specific change that occurs at 14 days, this is the duration of storage at which the largest effect on mortality and morbidity has been shown.

Other patient populations that have examined the influence of duration of storage of RBC on clinical outcomes include cardiac surgery and trauma patients. One of the largest studies to date is the retrospective analysis done by Koch et al., demonstrating that the storage of RBC for greater than 14 days lead to an increase in sepsis, intubation over 72 hours and in-hospital mortality. [13] Other studies have reported a similar interaction in cardiac patients. [14]–[17] Trauma patients often receive multiple blood transfusions and therefore highlight another group of patients in which storage of blood can be studied. The studies in this population are conflicting, but some reports do suggest an association between duration of storage of transfused RBC and adverse clinical outcomes [18]–[23].

The effect of duration of storage of transfused RBC on cancer patients has not been extensively studied. One study found no such effect on patients undergoing hematopoietic stem cell transplantation at one transplant centre. [24] The duration of storage of transfused RBC has been further studied in colorectal cancer patients as they often require transfusions due to gastrointestinal bleeding. Although one study showed an association between postoperative infections and older RBC units in these patients, [25] others have not been able to show a link between the blood “storage lesion” and clinical outcomes. [26] The association between duration of storage of transfused RBC and clinical outcomes is summarized in Table 1.

**Table 1:** Clinical Studies addressing Duration of Storage of Transfused Red Blood Cells and Patient Outcomes.

First Author, Year	Population		Number	Clinical Outcomes
**SIGNIFICANT ASSOCIATION**				
Martin 1994 [10]		Critical Care	698	Increased LOS
Purdy 1997 (11]		Critical Care	31	Increased mortality
Zallen 1999 [18]		Trauma	63	Increased multiorgan failure
Vamvakas 1999 [14]		Cardiac Surgery	416	Increased postoperative pneumonia
Mynster 2000 [25]		Colorectal Cancer	303	Increased overall infection rate
Offner 2002 [21]		Trauma	61	Increased infection rate
Keller 2002 [22]		Trauma	86	Increased LOS
Murrell 2005 [19]		Trauma	275	Longer ICU stay
Koch 2008 [13]		Cardiac Surgery	6,002	Increased in-hospital mortality and sepsis
Weinberg 2008 [20]		Trauma	1,813	Increased mortality
Spinella 2009 [23]		Trauma	202	Increased DVT and mortality
Eikelboom 2010 [15]		Cardiac Surgery	4,993	Increased mortality
Robinson 2010 [39]		Cardiology*	909	Increased 30 day mortality
Pettila 2011 [12]		Critical Care	757	Increased mortality
Sanders 2011 [17]		Cardiac Surgery	176	Increased postoperative LOS
Andreasen 2011 [16]		Cardiac Surgery	4,240	Increased postoperative infections
**NO ASSOCIATION**				
Edna 1998 [26]		Colorectal Cancer	446	Postoperative Infections
Vamvakas 2000 [40]		Cardiac surgery	268	LOS, ICU LOS, and length of intubation
Leal-Noval 2003 [41]		Cardiac surgery	897	ICU LOS, mechanical ventilation time, perioperative MI, postoperative infection
Hebert 2005 [42]		Cardiac Surgery/Critical Care	57	Mortality
Van der Watering 2006 [43]		Cardiac surgery	2,732	Mortality and ICU LOS
Taylor 2006 [44]		Critical Care	2,085	Nosocomial infections
Dessertaine 2008 [45]		Critical Care	534	Mortality
Yap 2008 [46]		Cardiac surgery	670	Mortality, renal failure, pneumonia, ICU LOS
Kekre 2011 [24]		HSCT	555	Mortality, LOS, organ toxicity
VanStraten 2011 [47]		Cardiac Surgery	5,316	Mortality
Katsios 2011 [48]		Critical Care	126	DVT
Dunn 2012 [49]		Liver transplant	509	Infection, organ failure, mortality

* Patients who underwent percutaneous coronary intervention;

LOS = length of stay; ICU = intensive care unit; DVT = deep vein thrombosis; MI = myocardial infarction; HSCT = hematopoietic stem cell transplantation.

There is however a well established connection between transfusion and poor clinical outcomes in a wide variety of patients. One review has summarized the adverse effect of transfusion on critical care, trauma and cardiac surgery patients, including the impact on infection rates, hospital length of stay and mortality. [8] Patients with a known malignancy often develop anemia, either related to their disease or treatment, thereby requiring blood transfusion. [27]–[29] For patients with an established diagnosis of cancer, studies suggest poorer OS if patients require a transfusion following tumour resection [30], [31].

We sought to investigate, within the framework of a large database, the influence of duration of storage of RBC transfusions on overall survival and cancer recurrence in patients diagnosed with cancer. Our secondary objective was to describe the transfusion practices in this large cancer centre database.

## Methods

### Ethics Statement

Data was collected from the Ottawa Regional Cancer Centre database and linked to data from The Ottawa Hospital Blood Bank, with approval from the Ottawa Hospital Research Ethics Board. Our study complies with the Declaration of Helsinki. We are required by the Public Hospitals Act to have a record of the patient’s care and treatment to be kept, meaning the health record. When this information is used for research, it is de-identified in an aggregate manner, as in this study. This is supported by the *Personal Health Information and Protection of Privacy Act* (PHIPA). Specifically for this study, as there was no patient contact or intervention, patient consent was not required by our local ethics board.

### Patients

The Ottawa Regional Cancer Centre (ORCC) is a tertiary cancer referral centre which maintains a database that prospectively collects cancer demographic and outcome data. This database was queried together with transfusion records from the Blood Bank at the Ottawa Hospital-General Campus. The hospital maintains transfusion records for all patients that have ever received a transfusion at the ORCC. Patients were eligible for inclusion in this study if they were diagnosed with any cancer between January 01, 2000 and December 31, 2005.

Data available from the ORCC database for analysis included patient age, gender, cancer type, stage, chemotherapy, radiation therapy and cancer-related surgery. Data available from the transfusion database included number of RBC units transfused by storage category. Units of blood were categorized as “new” if stored for less than 14 days, “intermediate” if stored for 14 to 28 days and “old” if stored for greater than 28 days. In the analysis of duration of storage of transfused RBC, only patients receiving exclusively one “aged” category of blood were included. For example, a patient who received both old (stored from greater than 28 days) and new (stored for less than 14 days) RBC units were excluded in the analysis of duration of storage of transfused RBC.

### Statistical Analysis

Statistical analysis was facilitated by SAS version 9.1. Baseline characteristics between the comparative groups were analyzed by ANOVA. Further, categorical variables and continuous variables were compared using Chi-squared and Wilcoxan rank-sum tests respectively. Kaplan-Meier analyses were used to examine differences in unadjusted survival while Cox-regression analyses were applied to adjust for potential confounding variables. Multivariable analyses were performed using a step-wise approach.

## Results

There were n = 27,591 patients diagnosed with any cancer at the ORCC between January 01, 2000 and December 31, 2005 with 1,929 (7.0%) patients receiving RBC transfusions within 1 year of the diagnosis of cancer. Baseline characteristics of those not transfused and transfused within the first year from diagnosis are summarized in Table 2. Of the patients transfused within the first year from diagnosis, 1335 (69.2%) received exclusively one “aged” category of RBC units.

**Table 2:** Baseline Patient Characteristics.

VARIABLE	NOT TRANSFUSED(N = 25,662)	TRANSFUSED(N = 1,929)	UNADJUSTEDP VALUE
**Age at diagnosis (mean years)**	63.3	63.4	0.74
**Gender(% Female)**	52.3%	46.7%	<0.0001
**Type of Cancer: n (%)**			
** Gastrointestinal**	4,323 (16.9%)	477 (24.7%)	<0.0001
** Breast**	5,141 (20.0%)	119 (6.2%)	
** Genitourinary**	4,246 (16.6%)	166 (8.6%)	
** Lung**	3,419 (13.3%)	455 (23.6%)	
** Hematologic**	1,347 (5.3%)	183 (9.5%)	
** Other**	7,186 (28.0%)	529 (27.4%)	
**Stage: n (%)**			
** 0**	646 (2.5%)	6 (0.3%)	<0.0001
** I**	3,813 (14.9%)	100 (5.2%)	
** II**	4,612 (18.0%)	203 (10.5%)	
** III**	2,538 (9.9%)	321 (16.6%)	
** IV**	2,583 (9.9%)	570 (29.5%)	
** Unknown**	11470 (44.7%)	729 (37.8%)	
**Treatment: n (%)**			
** Chemotherapy**	12,088 (47.1%)	1,248 (64.7%)	0.045
** Radiation**	14,250 (55.5%)	1,249 (64.7%)	0.078
** Surgery**	14,666 (57.2%)	763 (39.6%)	0.59
**Cancer Recurrence: n (%)**			
	8,533 (33.3%)	1,092 (56.6%)	<0.0001

The mean number of RBC transfusions was 3.42 (95% CI 0.22, 6.62) with the majority of patients (55.5%) receiving between 1–2 RBC units. Of those patients who received a RBC transfusion within 1 year from diagnosis, most were transfused within the first 6 months (56.8%). Proportionately more male patients than female patients (53.3% versus 46.7% respectively, p<0.0001) required a transfusion within the first year from cancer diagnosis. There was a significant difference amongst patients with different malignancies requiring RBC transfusion (p<0.0001). Almost half of the patients requiring transfusion within the first year from diagnosis (48.3%) had either a gastrointestinal or lung malignancy. The stage of cancer was not different amongst patients who were and were not transfused (Table 2). Table 3 summarizes RBC transfusions received by our study population by cancer type. Patients who received chemotherapy were more likely to receive a RBC transfusion (p = 0.05). There was, however, no effect of patients undergoing radiation or cancer-related surgery on RBC transfusion requirements (p = 0.08 and p = 0.59 respectively). The duration of storage of RBC transfused by cancer type was not significantly different (p = 0.09) (Table 4).

**Table 3:** Number of Red Blood Cell Transfusions by Cancer Type.

	Number of Units Transfused N (%)			
	1–2	3–5	6+	Total
**Gastrointestinal**	278 (58.3%)	136 (28.5%)	63 (13.2%)	477
**Breast**	79 (66.4%)	30 (25.2%)	10 (8.4%)	119
**Genitourinary**	92 (55.4%)	45 (27.1%)	29 (17.5%)	166
**Lung**	274 (60.2%)	124 (27.3%)	57 (12.5%)	455
**Hematology**	73 (39.9%)	68 (37.2%)	42 (23.0%)	183
**Other**	293 (55.4%)	139 (26.3%)	97 (18.3%)	529
**Total**	1,089	542	298	1,929

**Table 4:** Duration of Storage of Transfused Red Blood Cells by Cancer Type.

	Age of Blood Transfused			
	New	Intermediate	Old	Total
**Gastrointestinal**	98 (27.8%)	175 (49.6%)	80 (22.7%)	353
**Breast**	22 (25.6%)	41 (47.7%)	23 (26.7%)	86
**Genitourinary**	36 (32.4%)	57 (51.4%)	18 (16.2%)	111
**Lung**	71 (22.0%)	186 (57.7%)	66 (20.4%)	323
**Hematology**	21 (20.0%)	60 (57.1%)	24 (22.9%)	105
**Other**	112 (31.4%)	172 (48.2%)	73 (20.5%)	357
**Total**	360	691	284	1,335

**Figure 1:**
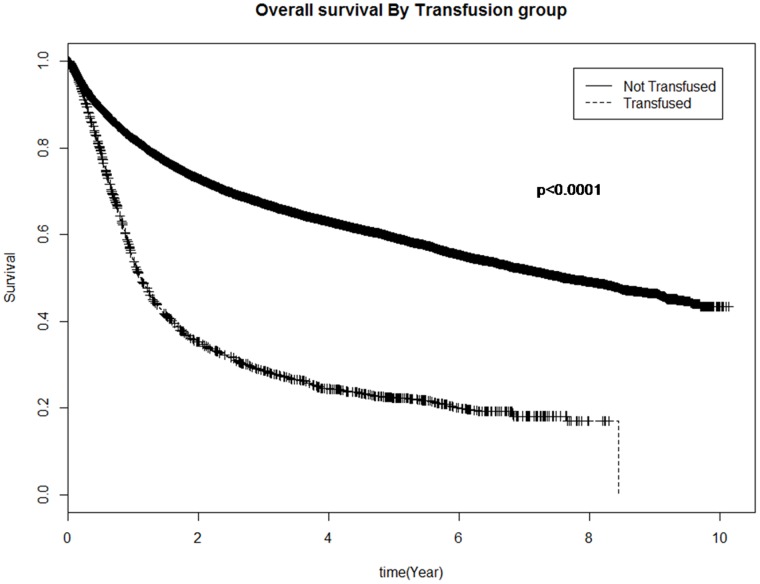
Overall survival of cancer patients.

**Figure 2:**
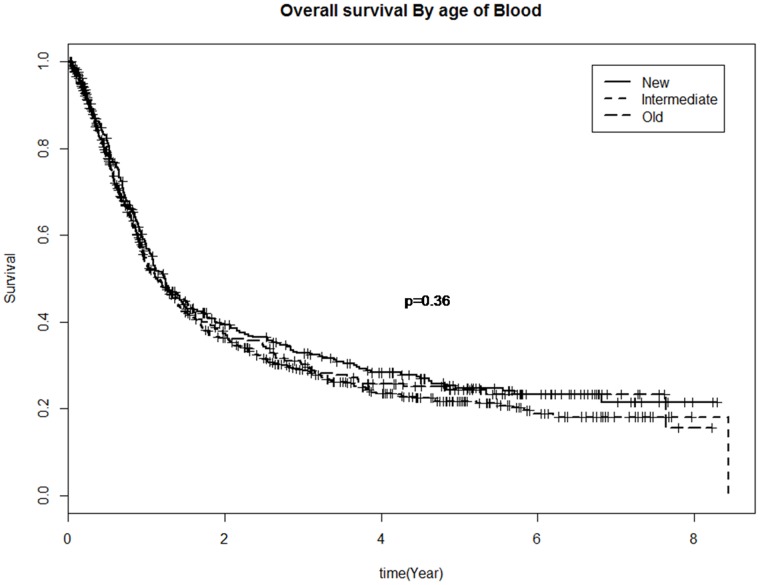
Overall survival by duration of storage of transfused red blood cells.

The median OS was inferior for patients who were transfused versus those not transfused (1.1 vs 7.5 years respectively; p<0.0001, Figure 1). The number of RBC units transfused also significantly influenced OS (median OS for 1–2 units was 1.2 years, 3–5 units was 1.05 years and 6 or more units was 0.9 years; p = 0.0017) in univariate analysis. The duration of storage of transfused RBC units was not, however, associated with OS where the median survival for only new, only intermediate and only old RBC units transfused was 1.2, 1.7, and 1.1 years respectively (p = 0.36, Figure 2).

**Figure 3:**
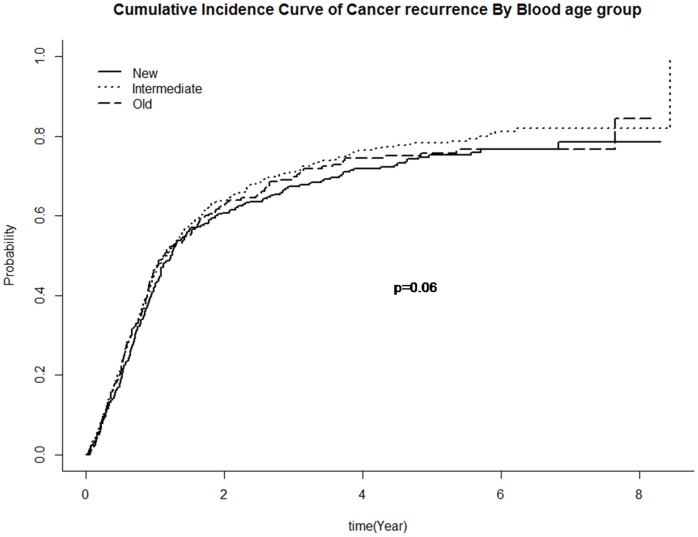
Time to cancer recurrence by duration of storage of transfused red blood cells.

Cancer recurrence, defined as recurrence of original malignancy or new metastatic disease, was significantly higher in patients who received a RBC transfusion than those who did not (56.3% versus 33.0% respectively; p<0.0001). However, recurrence rates were not significantly influenced by the duration of storage of transfused RBC (56.8% for only “new”, 58.7% for only “intermediate” and 50.5% for only “old” RBC units transfused; p = 0.06). Time to cancer recurrence was also not influenced by the duration of storage of transfused RBC with a median time to recurrence for only “new”, “intermediate” and “old” blood of 0.50, 0.35 and 0.50 years respectively (p = 0.06, Figure 3).

We performed a multivariate analysis to determine the impact of the following variables on OS: age, gender, cancer type, stage, chemotherapy, radiation, cancer-related surgery, cancer recurrence, number and duration of storage of RBC units transfused. Patient age as a continuous variable was significantly associated with OS (p = 0.0099). Amongst all cancers, lung cancer was associated with inferior OS (p<0.0001). Stage of disease was analyzed as a categorical variable, with advanced disease being defined as stage 3 or 4. Advanced stage, chemotherapy, radiation, cancer-related surgery and cancer recurrence were also associated with inferior OS (p<0.0001, p<0.0001, p = 0.0011, p<0.0001, and p = 0.0086 respectively). However, neither the number of units of RBC transfused nor the duration of storage of transfused RBC was associated with OS. In order to ascertain that cancer recurrence was not the key modifier of the effects on OS, diluting out the effects of other variables, we repeated our multivariate analysis without cancer recurrence as a variable. In this analysis, there was still no association between OS and the number of units of RBC transfused or the duration of storage of transfused RBC. Similarly, patient age (p = 0.01), advanced stage (p<0.0001), lung cancer (p<0.0001), chemotherapy (p<0.0001), radiation (p = 0.002) and cancer-related surgery (p<0.0001) were significantly associated with OS.

We considered cancer recurrence as a separate clinical outcome, and evaluated similar variables as that analyzed for OS. In a multivariable analysis, patient age (p = 0.006), advanced stage (p<0.0001) and lung cancer (p<0.0001) were associated with cancer recurrence. Further, transfusion of more than 6 units of RBC compared with 2 or less units of RBC was associated with an increased risk of cancer recurrence (p = 0.022). However, the duration of storage of transfused RBC was not associated with risk of cancer recurrence.

## Discussion

The data from our study suggests that the duration of storage of transfused RBC has a negligible effect on overall survival (OS) of cancer patients. Not surprisingly, OS was influenced by the patient’s age, advanced stage, chemotherapy and radiation use, cancer-related surgery and cancer recurrence. Although receiving a RBC transfusion was associated with OS in univariate analysis, this was not evident when accounting for confounding variables. Cancer recurrence was influenced by transfusion requirement, but both cancer recurrence rate and time to recurrence were not significantly influenced by duration of storage of RBC transfusion. We conclude, therefore, that the duration of storage of transfused RBC units does not influence OS or cancer recurrence in patients with underlying malignancy.

Our findings are in contrast to results in other populations, particularly surgical patients, who are traditionally highly transfused (Table 1). In non-surgical populations, patients with malignancy represent an increasing group of patients requiring transfusions, given the increasing use of myelosuppressive therapy, oncologic surgery and underlying disease process. Bone marrow involvement of malignancy and treatment related anemia may contribute to the necessity for transfusion in these patients. Notably, amongst patients diagnosed with cancer in a five year time period, only 7% required RBC transfusion during the first year of diagnosis. More than half of these patients had lung or gastrointestinal (GI) cancer in our study. This is similar to previous studies that have shown a high rate of RBC transfusion amongst patients with lung [27] and GI cancer. [28] Patients with lung cancer are often hypoxic and may potentially benefit from RBC transfusion while patients with a GI malignancy often present with GI bleeding or require surgical intervention, two risk factors for anemia. In contrast, patients with hematological malignancies did not appear to require more RBC transfusions compared to other malignancies despite a belief that that they may receive more cytotoxic treatments and have the potential for more involvement of the bone marrow. This observation may have been influenced by the pattern of care for patients with acute leukemia and multiple myeloma at our centre. These patients are assessed directly by Hematologists at our centre, where clinical data is not often well documented within the ORCC database.

It is not surprising that RBC transfusions are associated with OS of cancer patients in our study. It remains unclear, however, whether RBC transfusions contribute to adverse outcomes or whether transfusion is a surrogate for patients at high risk of adverse outcomes. [32] Consequently, we cannot determine whether RBC transfusion contributes to mortality in our observational study. Nonetheless, our results suggest that transfused cancer patients requiring chemotherapy, radiation and surgery had a higher mortality, while the extent of RBC transfusion and the duration of storage of transfused units were not associated with OS in multivariate analysis. We suspect that patients’ underlying disease and condition, rather than RBC transfusions, determined OS.

There is evidence to suggest that RBC transfusion may have a negative impact on cancer recurrence by mediating an immunosuppressive mechanism that facilitates the propagation of malignant cells. [33] This immune-mediated mechanism of cancer progression may be largely due to immune dysregulation in the cancer patient, particularly in individuals that require transfusion. [34] Components of the RBC transfusion that might modulate a patient’s immune system include cell debris that accumulates during RBC storage as well as the presence of leukocytes in the RBC unit. [34] Most studies that reported an association between cancer recurrence and storage of RBC [34] were conducted prior to universal leukoreduction (which is standard practice in Canada since 2000). In the era of universal leukoreduction, our study suggests that any immunomodulatory effect caused by prolonged storage of transfused RBC units on cancer recurrence is not significant. This is in agreement with a recent report in prostate cancer patients who were followed for recurrence after prostatectomy. [35] Interestingly, the duration of storage of blood for less than 21 days may be a risk factor for cancer recurrence in patients with colorectal cancer undergoing surgery [36].

There are limitations to our study that warrant attention, predominantly associated with our retrospective design. Firstly, our results may be subjected to bias, incomplete information or misdiagnosis. We attempted to minimize selection bias by including all consecutive patients assessed by the ORCC where the underlying malignancy has been confirmed by pathology. Secondly, while our ORCC database provides a date of cancer diagnosis, we could not ascertain the timing of any associated chemotherapy, radiation or cancer-related surgery. In the absence of temporal trends, the influence of RBC transfusion and duration of storage of transfused RBC cannot be fully ascertained. Thirdly, potential confounders and effect modifiers could not be adequately assessed. For instance, specific cancer related prognostic factors, such as hormone receptor status in breast cancer, were not available. Rather, the global severity of the underlying cancer was assessed by the American Joint Committee on Cancer TNM staging. Further, clinical outcomes may be affected by factors such as initial response to chemotherapy, time to first treatment, and use of erythropoietin stimulating agents, all of which were unfortunately not available within the context of this large database study. Despite these limitations, to our knowledge, this is one of the largest studies to examine the influence of duration of storage of transfused RBC on cancer patient outcomes. We have previously published a similar analysis in patients undergoing hematopoietic stem cell transplantation where, in concordance with our present study, the number of RBC transfusions but not the duration of storage of RBC units impacted clinical outcomes. [24] Finally, our results can be interpreted as similar to previously published prospective cohorts (Table 1). Ultimately, the effects of duration of storage of transfused RBC can only be adequately assessed within the context of prospective randomized trials. Indeed, there are several that are ongoing to address the influence of duration of storage of transfused RBC in cardiac surgery (NCT00458783, NCT00991341), premature infants (NCT00326924 [37]), critical care (NCT01638416, ISRCTN44878718 [38]), hospital inpatients (ISRCTN38768001) and hematology patients (ISRCTN06273643).

In summary, our study highlights the inferior survival of cancer patients requiring RBC transfusions; however, there is no apparent influence of the duration of storage of transfused RBC units on OS or cancer recurrence. We conclude that current RBC transfusion policies that do not differentiate between duration of storage of RBC units are adequate for patients with underlying malignancy.
